# PAQR3 expression is downregulated in human breast cancers and correlated with HER2 expression

**DOI:** 10.18632/oncotarget.3657

**Published:** 2015-03-26

**Authors:** Zhenghu Li, Zhi-Qiang Ling, Weiwei Guo, Xiao-Xiao Lu, Yi Pan, Zhenzhen Wang, Yan Chen

**Affiliations:** ^1^ Key Laboratory of Nutrition and Metabolism, Institute for Nutritional Sciences, Shanghai Institutes for Biological Sciences, Chinese Academy of Sciences, Graduate School of the Chinese Academy of Sciences, Shanghai, China; ^2^ Zhejiang Cancer Research Institute, Zhejiang Province Cancer Hospital, Zhejiang Cancer Center, China

**Keywords:** breast cancer, PAQR3, HER2, survival, cell proliferation

## Abstract

PAQR3 is a newly discovered tumor suppressor and its functional role in breast cancer has not been well characterized. We report here that PAQR3 is associated with the progression and survival of human patients with breast cancer, as well as cell proliferation and migration of human breast cancer cells. PAQR3 mRNA level was robustly downregulated in human breast cancer samples compared with their corresponding para-cancerous histological normal tissues (*n* = 82, *P* < 0.0001). The mRNA level of PAQR3 was negatively correlated with HER2 expression (*P* < 0.0001) and disease-free survival of the patients (*P* < 0.0001). PAQR3 overexpression inhibited cell proliferation, colony formation and migration of breast cancer cells including MCF7, SKBR3, MDA-MD-231, MDA-MD-468 and MDA-MD-453 cells. Knockdown of PAQR3 in MDA-MD-231 cells elevated cell proliferation and migration. Inhibition of HER2 by trastuzumab increased PAQR3 expression in SKBR3 cells. In conclusion, PAQR3 expression is frequently downregulated in human breast cancers inversely correlated with HER2 expression. PAQR3 is able to modulate the proliferation and migration of breast cancer cells. Our data indicate that PAQR3 functions as a tumor suppressor in the development of human breast cancers.

## INTRODUCTION

Breast cancer is one of the most common cancers worldwide. Risk factors for developing breast cancer include obesity, lack of physical exercise, drinking alcohol, hormone replacement therapy during menopause, ionizing radiation, early age at first menstruation, and having children late or not at all. Breast cancer is commonly treated with various combinations of surgery, radiation therapy, chemotherapy, and hormone therapy [1]. The outcomes for breast cancer vary depending on the cancer type, extent of disease and the age of the patients. The survival rates in the developed countries are high, with between 80% and 90% of those in England and the United States alive for at least 5 years. The survival rate in developing countries is relatively poor. Breast cancer cells bear (noun-verb agreement) different receptors on their surface and in their cytoplasm or nucleus, among these receptors the important ones are estrogen receptor (ER), progesterone receptor (PR), and human epidermal growth factor receptor 2 (HER2) [2]. These receptors especially HER2 have served as targets for treatment of breast cancers [3]. It was noted that the mitochondrial pathway for apoptosis is important for death induced by HER2 inhibition [4].

HER2 is a major player in the proliferation and survival of cancer cells and about 20% breast cancers are addicted to HER2 [5, 6]. HER2 expression in the cancer cells is achieved by overexpression of the HER2 protein, amplification of the *HER2* gene, or activating mutations of the gene [6]. HER2-positive breast cancers are characterized by poor clinical prognosis and aggressive tumor behavior [7, 8]. The HER2 addiction of breast cancers is best demonstrated by the outstanding benefit of HER2-targeting therapy such as the monoclonal antibody trastuzumab (herceptin) for the treatment of HER2-positive breast cancer patients. However, the heterogeneous sensitivity to HER2-targeting therapy has indicated that targeting to other genetic alterations/pathways and immune modulation may pave the way for future treatment of the patients who are resistant to HER2-targeting therapy [9]. Among the signaling pathways initiated by HER2, PI3K/Akt is considered as the most important survival pathway downstream of HER2-HER3 dimerization [10]. A number of preclinical studies indicate that resistance to HER2-targeting therapy can be overcome by inhibition of the PI3K/Akt pathway [11, 12].

PAQR3, also named as RKTG (Raf kinase trapping to Golgi), belongs to the PAQR (progesterone and adipoQ recepotor) superfamily [13]. It is a type 3 membrane protein specifically localized in the Golgi apparatus with 7 trans-membrane domains [14]. PAQR3 was originally characterized as a spatial regulator of Raf kinase [15]. PAQR3 binds Raf kinase and sequesters it to the Golgi apparatus and consequently represses Ras/MAPK signaling [15]. PAQR3 functions as a tumor suppressor mainly due to its inhibitory activity on Raf/MAPK and PI3K/Akt signaling pathways [15-19]. PAQR3 has a negative effect on cell proliferation, migration, sprouting, and angiogenesis of endothelial cells [20]. PAQR3 has a functional interaction with p53 in cancer formation and epithelial-mesenchymal transition (EMT) [18]. PAQR3 has a suppressive function in A375 human melanoma cells that harbor an oncogenic B-Raf mutation V600E, the most common mutation in melanoma [17]. PAQR3 also has a suppressive activity in chemical carcinogen-induced mitogenesis and tumor formation in mouse skin [16]. Genetic depletion of PAQR3 in mice is able to enhance intestinal tumor formation under the genetic background of heterozygous mutation of tumor suppressor adenomatous polyposis coli (APC) [21]. Lately, it was found that PAQR3 is frequently downregulated in human gastric and liver cancers [22, 23]. In this study, we investigated the potential functions of PAQR3 in human breast cancers.

## RESULTS

### PAQR3 is significantly downregulated in primary breast cancer tissues and correlated with HER2 expression

**Figure 1:**
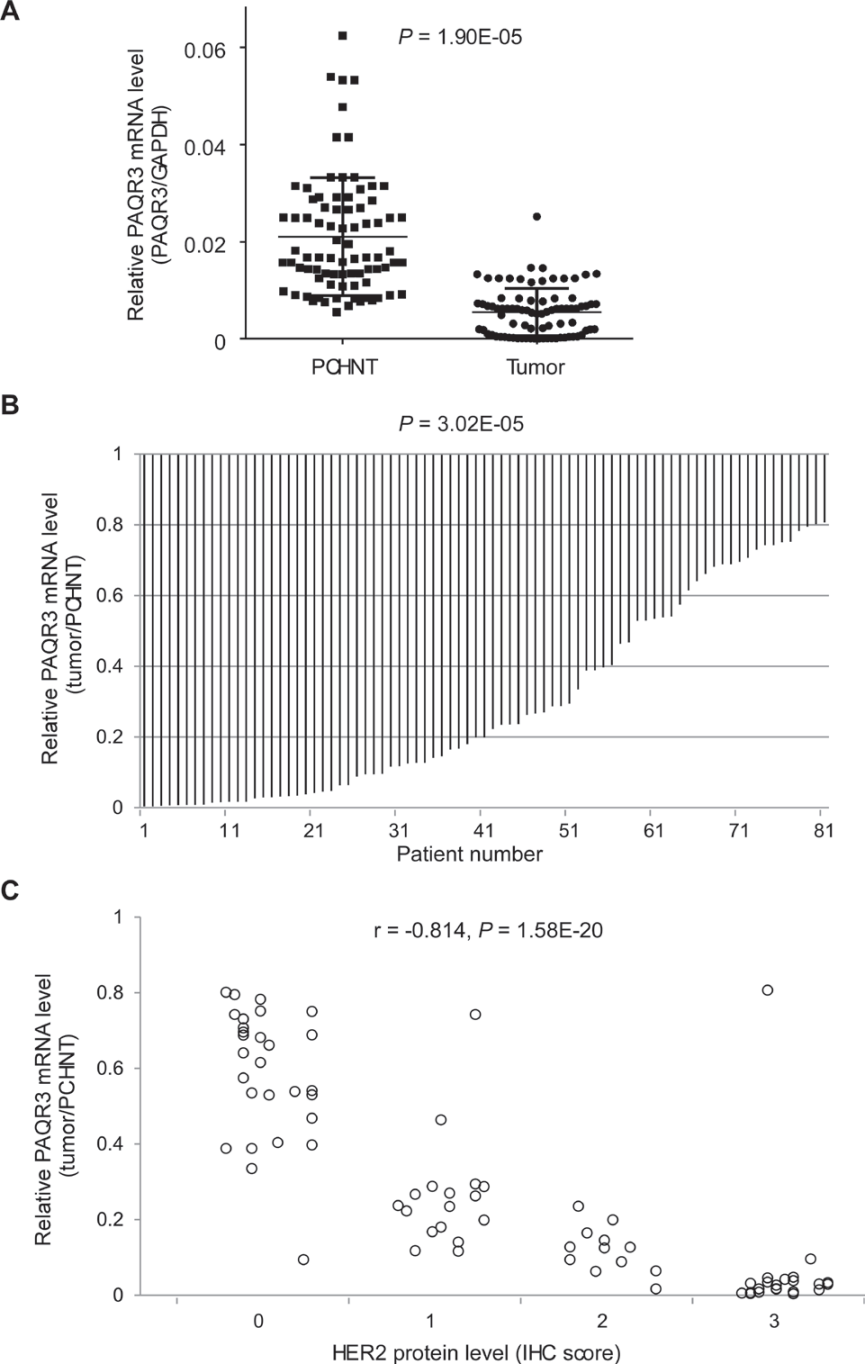
Expression of PAQR3 is reduced in human breast cancer samples and inversely correlated with HER2 expression The relative level of PAQR3 mRNA in human breast cancer tissues in comparison with PCHNT specimen from 82 breast cancer patients is shown in (**A**) PAQR3 mRNA level was determined by quantitative RT-PCR and adjusted for GAPDH. The relative ratio of PAQR3 mRNA in the cancer samples *vs* PCHNT samples is shown in (**B**). The tumor/PCHNT ratio < 0.5 is considered as low expression. The correlation of PAQR3 mRNA level with HER2 expression is shown in **(C)**. IHC was used to determine the protein expression level of HER2. The IHC score of HER2 is plotted with the mRNA level of PAQR3. Note that PAQR3 expression level is inversely correlated with HER2 expression level.

To investigate the potential role of PAQR3 as a suppressor in human breast cancers, we characterized the expression status of PAQR3 transcript in 82 patients with primary breast cancer. The mRNA level of PAQR3 was determined in both the primary breast cancer samples together with their corresponding para-cancerous histological normal tissue (PCHNT). Intriguingly, we found that PAQR3 mRNA level was robustly reduced in most of the cancer samples compared to PCHNT. Using the standard of over two-fold change, PAQR3 mRNA was significantly lower in 58 tumors (70.7%, *P* < 0.0001) than the paired PCHNT samples (Figure 1A and 1B, Table 1). The mRNA level of PAQR3 was also associated with a few clinical characteristics of the patients (Table 1). The expression status of PAQR3 in the breast cancer samples was strongly correlated with the differentiation of the tumor (*P* < 0.0001), with low expression of PAQR3 associating with poor differentiation of the tumor. In addition, PAQR3 expression level was slightly associated with TNM stage (*P* = 0.044), with lower PAQR3 mRNA level associating with higher TNM stage.

One of most interesting discoveries was that the PAQR3 mRNA level was closely correlated with the status of HER2 expression (γ = −0.814, *P* < 0.0001). Low expression of PAQR3 was tightly associated with high expression of HER2 (Figure 1C). As shown in Table 1, in 29 HER2-negative tumors, 6 of them had reduction of PAQR3 expression (21%). However, in 53 HER2-positive tumors, 52 of them had a decrease in PAQR3 expression (98%). However, the expression level of PAQR3 was not associated with the status of ER expression or PR expression (Table 1). Collectively, these data not only pinpoint that PAQR3 expression level is markedly altered in human breast cancers, but also suggest that such change is closely associated with HER2 expression in the tumors.

**Table 1:** Correlation between relative PAQR3 expression level and clinicopathologic parameters of the patients

Variables		n	PAQR3 mRNA level		χ^2^
			Low	Normal/High	(*P*-value)
Age (years)					
	<50	50	39	11	3.270
	≥50	32	19	13	(0.071)
Tumor location					
	Left	44	31	13	0.004
	Right	38	27	11	(0.953)
Tumor size					
	≤2 cm	39	27	12	0.081
	>2 cm	43	31	12	(0.776)
Differentiation					
	Moderate/high	34	11	23	41.326
	Poor	48	47	1	(1.289E-10)
T stage					
	I/II	67	47	20	0.060
	III/IV	15	11	4	(0.806)
Lymph node metastasis					
	Negative	38	24	14	1.962
	Positive	44	34	10	(0.161)
TNM stage					
	I/II	55	35	20	4.062
	III/IV	27	23	4	(0.044)
ER status					
	Negative	44	30	14	0.298
	Positive	38	28	10	(0.585)
Her2 status					
	Negative	29	6	23	54.274
	Positive	53	52	1	(1.744E-13)
PR status					
	Negative	42	35	14	0.029
	Positive	39	23	10	(0.866)

Note: n, number of patients; ER, estrogen receptor; PR, progesterone receptor; Her2, human epidermal growth factor receptor 2; TNM, tumor, node, metastasis. Fisher's exact test was used for statistical analyses. PAQR3 mRNA low expression is defined as tumor/PCHNT < 0.5 and normal/high expression as tumor/PCHNT >= 0.5.

### The expression level of PAQR3 is associated with the prognosis of the breast cancer patients

**Figure 2:**
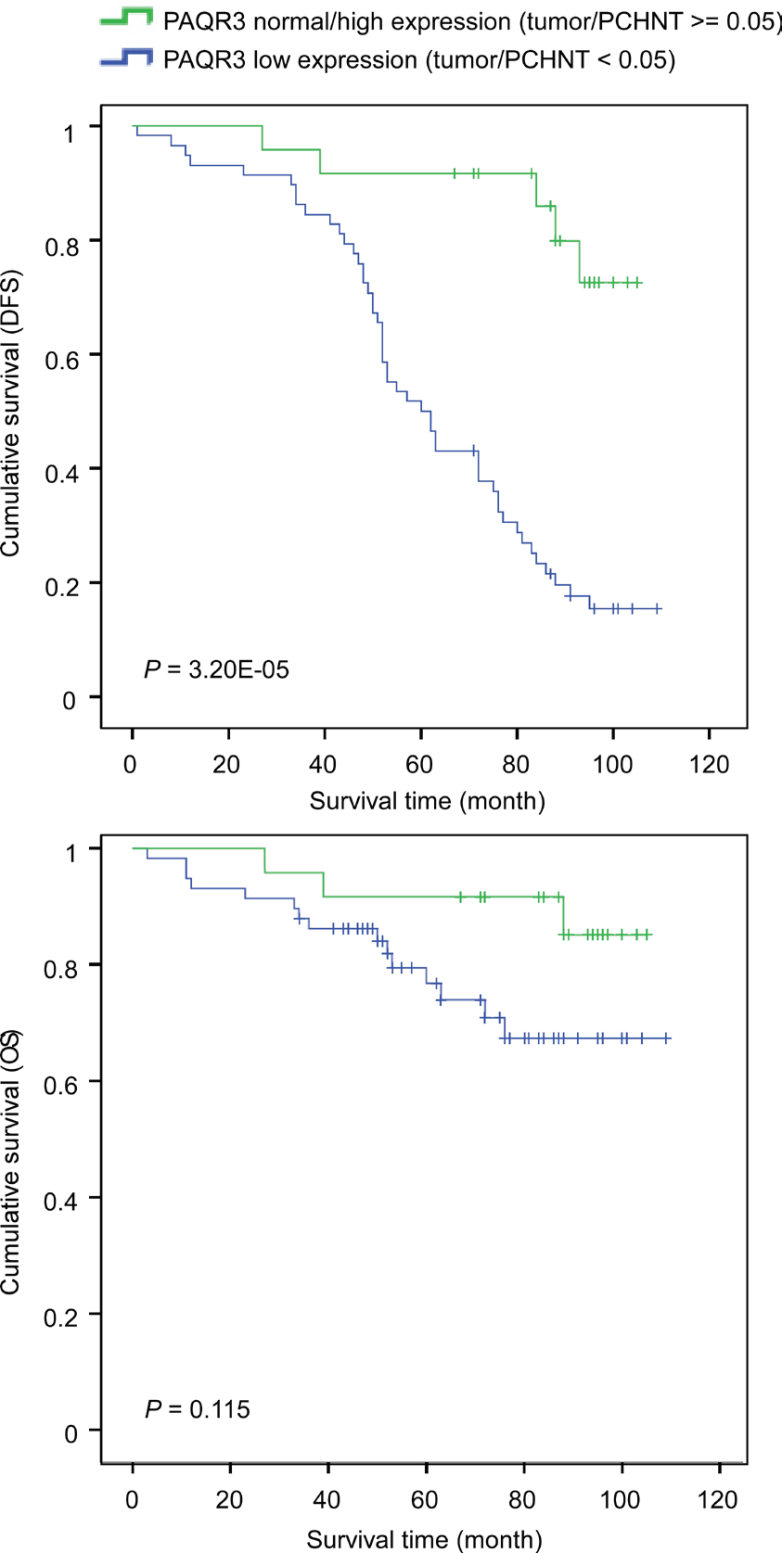
Correlation of PAQR3 expression level with survival of breast cancer patients Kaplan-Meier curves of disease-free survival (DFS, shown in A) and overall survival (OS, shown in B) in post-surgery patients with breast cancers according to the expression level of PAQR3.

We next investigated the association of PAQR3 expression level with the survival of the patients. Both disease-free survival (DFS) and overall survival (OS) were determined. DFS was defined as the time from surgery to first recurrence and OS was the time from surgery to death. We found that the average duration of DFS in patients with PAQR3 downregulation in the tumors was significantly shorter than those with normal to high expression of PAQR3 in the tumors (Figure 2 and Table 2). The average DFS in patients with low expression of PAQR3 was 64.1 months *vs* 95.9 months in patients with normal to high expression of PAQR3 (Table 2, *P* < 0.0001). However, the average OS was only slightly different between the patients with low expression of PAQR3 and those with normal to high expression of PAQR3 (Table 2, *P* = 0.081). This was likely due to the fact that we only analyzed the patient survival for about 5 years and most patients were still alive due to effective therapy. In addition, we observed that a few other previously characterized clinical parameters were associated with patient survival (Table 2), such as lymph node metastasis (*P* = 0.021) and TNM stage (*P* = 0.043). Collectively, these data indicate that the expression level of PAQR3 might serve as an independent marker to predict the survival of the breast cancer patients.

**Table 2:** Univariate and multivariate analyses of survival of 82 patients with breast cancers

			DFS					OS				
		n	Survival	Univariate analysis		Multivariate analysis		Survival	Univariate analysis		Multivariate analysis	
			(months)	χ^2^	*P*	HR (95% CI)	*P*	(months)	χ^2^	*P*	HR (95% CI)	*P*
Age (years)												
	<50	50	74.2	0.026	0.873	1.217（0.453-3.268）	0.697	92.6	0.004	0.948	1.440（0.797-2.603）	0.227
	≥50	32	71.8					88.5				
Tumour size												
	≤2 cm	39	79.4	2.812	0.094	1.549（0.587-4.090）	0.377	95.4	1.022	0.312	1.530（0.870-2.693）	0.140
	>2 cm	43	67.6					86.0				
T stage												
	I/II	67	74.1	0.077	7.821E-01	0.636（0.155-2.609）	0.530	92.9	0.014	0.906	0.522（0.226-1.208）	0.129
	III/IV	15	71.2					85.9				
Lymph node metastasis												
	Negative	38	83.6	5.317	0.021	1.223（0.384-3.893）	0.733	97.7	1.149	0.284	1.553（0.799-3.016）	0.194
	Positive	44	64.7					84.4				
TNM stage												
	I/II	55	80.1	4.094	0.043	1.490（0.414-5.360）	0.542	96.3	1.252	0.263	1.321（0.631-2.768）	0.460
	III/IV	27	60.1					81.5				
PAQR3												
	Normal/high	24	95.9	22.258	2.383E-06	2.776（0.760-10.133）	0.122	97.9	3.042	0.081	7.334（2.839-18.947）	3.870E-05
	Low	58	64.1					88.0				

Note: PAQR3 mRNA low expression is defined as tumor/PCHNT < 0.5 and normal/high expression as tumor/PCHNT >= 0.5.

### PAQR3 regulates cell proliferation of breast cancer cell lines

**Figure 3:**
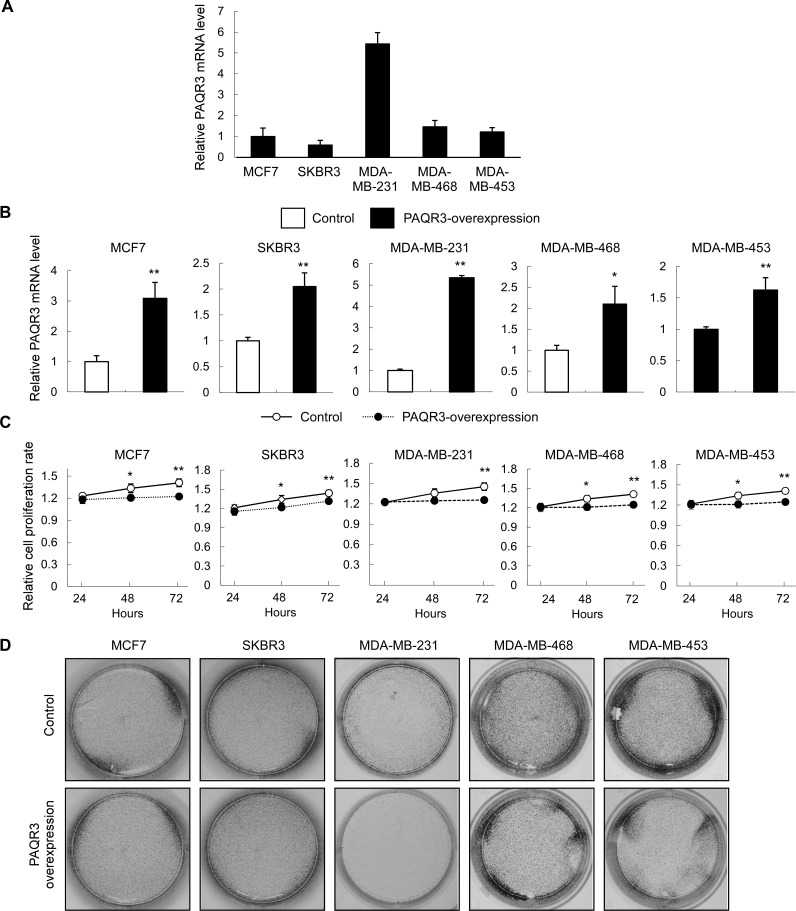
PAQR3 overexpression reduces cell growth and colony formation of human breast cancer cells (**A**) The expression level of PAQR3 in breast cancer cells were detected by quantitative RT- PCR. (**B**) The expression levels of PAQR3 in control and PAQR3-overexpressing cells by lentivirus infection were detected by quantitative RT-PCR. (**C**) MTT assay was used to determine the cell proliferation rate with the cells with or without overexpression of PAQR3 at the time point as indicated. The data are shown as mean ± SD and * for *P* < 0.05 and ** for *P* < 0.01. (D) Colony formation assay was performed with these cells. The cells were seeded into 6-well plates and cultured for 5 days, followed by crystal violet staining.

We next investigated whether PAQR3 has a direct effect on the growth and migration of breast cancer cells. We used five breast cancer cell lines with different status of HER2 expression. MCF7 is an ER-positive breast cancer cell line. SKBR3, MDA-MD-468 and MDA-MD-453 cell lines are HER2-positive. MDA-MD-231 cell line is triple-negative. We analyzed the PAQR3 expression levels of these cell lines (Figure 3A). Consistent with the clinical data that HER2 expression is inversely correlated with PAQR3 expression, we found that the HER2-negative MDA-MD-231 cells had the highest level of PAQR3 expression (Figure 3A).

We next established five breast cancer cell lines with stable expression of PAQR3 using lentivirus infection. Overexpression of PAQR3 in these cells was confirmed by quantitative RT-PCR (Figure 3B). The cell proliferation rate of these cells was investigated by MTT and colony formation assays. As shown in Figure 3C, the cell growth rate was significantly reduced by PAQR3 overexpression. Colony formation was used to investigate the potential of tumorigenesis of the breast cancer cells. We found that PAQR3 overexpression profoundly reduced colony formation in MDA-MD-231, MDA-MD-468 and MDA-MD-453 cells (Figure 3D). Collectively, these data indicate that PAQR3 has a negative effect on the growth of breast cancer cells.

### PAQR3 negatively regulates migration of breast cancer cells

**Figure 4:**
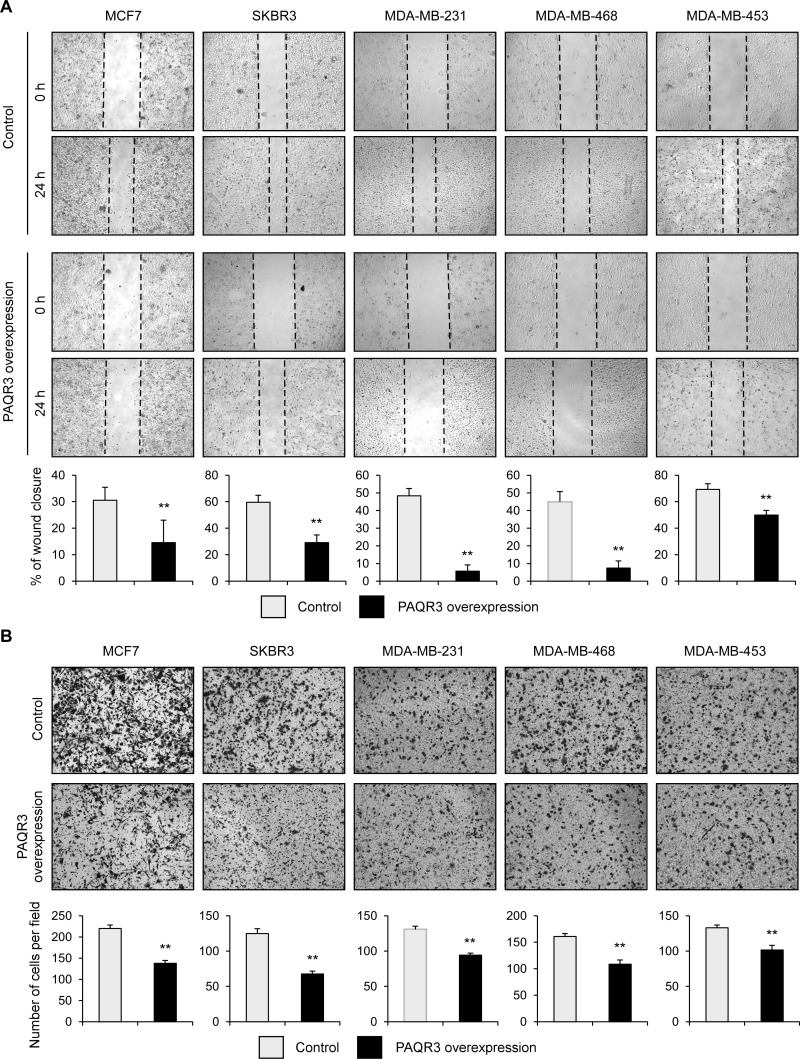
PAQR3 overexpression decreases migration of breast cancer cells (**A**) A scratched-wound healing assay was performed with the cells expressing control or PAQR3-expressing plasmids after lentivirus infection, followed by photography at 0 h and at 24 h after the scratch. The statistical results are shown in the lower panel. (**B**) A transwell assay was performed with the cells, followed by photography and counting. The lower panel denotes the statistical results. All the data are shown as mean ± SD and ** for *P* < 0.01.

We also analyzed the potential role of PAQR3 on the migratory activity of breast cancer cells. The wound healing assay revealed that overexpression of PAQR3 could significantly reduce the migration rate of all five breast cancer cell lines (Figure 4A). Consistently, the transwell assay also confirmed that the cell migration rate was significantly decreased by PAQR3 overexpression in these breast cancer cells (Figure 4B). These data, therefore, indicate that in addition to the regulatory function on cell proliferation, PAQR3 has an impact on the migration of breast cancer cells.

### Knockdown of PAQR3 in MDA-MB-231 cells increases cell proliferation and migration

**Figure 5:**
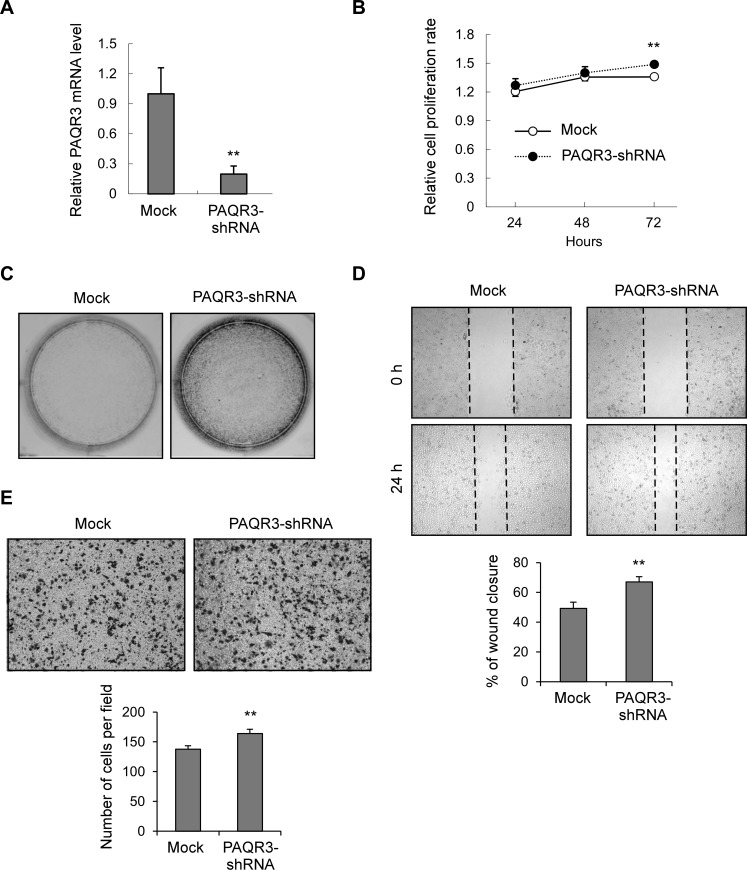
Knockdown of PAQR3 in MDA-MB-231 cells enhances cell growth and migration (**A**) The mRNA level of PAQR3 in MDA-MB-231 cells infected with lentivirus containing mock or PAQR3-specific shRNA as detected by quantitative RT-PCR. (**B**) Cell proliferation rate of these cells were determined by MTT assay at the indicated time point. (**C**) Colony formation assay was performed in these cells that were stained by crystal violet. (**D**) A scratched-wound healing assay was performed with these cells, followed by photography at 0 h and 24 h after the scratch. The lower panel denotes statistical analysis. (**E**) Transwell assay was performed with these cells, followed by photography and counting. The statistical results are shown in the lower panel. All the data are shown as mean ± SD and ** for *P* < 0.01.

As shown in Figure 3A, MDA-MB-231 cell line had the highest expression level of PAQR3 among the five breast cancer cell lines. We further analyzed the function of PAQR3 by establishing a MDA-MB-231 cell line with downregulation of PAQR3 by lentivirus infection. A control shRNA or PAQR3-specific shRNA was introduced into these cells. As confirmed by quantitative RT-PCR (Figure 5A), the mRNA level of PAQR3 was successfully reduced by a PAQR3-specific shRNA as previously reported by us [20]. Knockdown of PAQR3 could upregulate cell proliferation rate of MDA-MB-231 cells by MTT assay (Figure 5B). The capacity of colony formation was also enhanced by PAQR3 knockdown (Figure 5C). Consistently, the migration ability of MDA-MB-231 cells was significantly elevated by PAQR3 downregulation by both the wound healing assay (Figure 5D) and the transwell assay (Figure 5E). These data, therefore, further confirmed the negative effect of PAQR3 on the proliferation and migration of human breast cancer cells.

### PAQR3 expression is enhanced by inhibition of HER2 in SKBR3 cells

**Figure 6:**
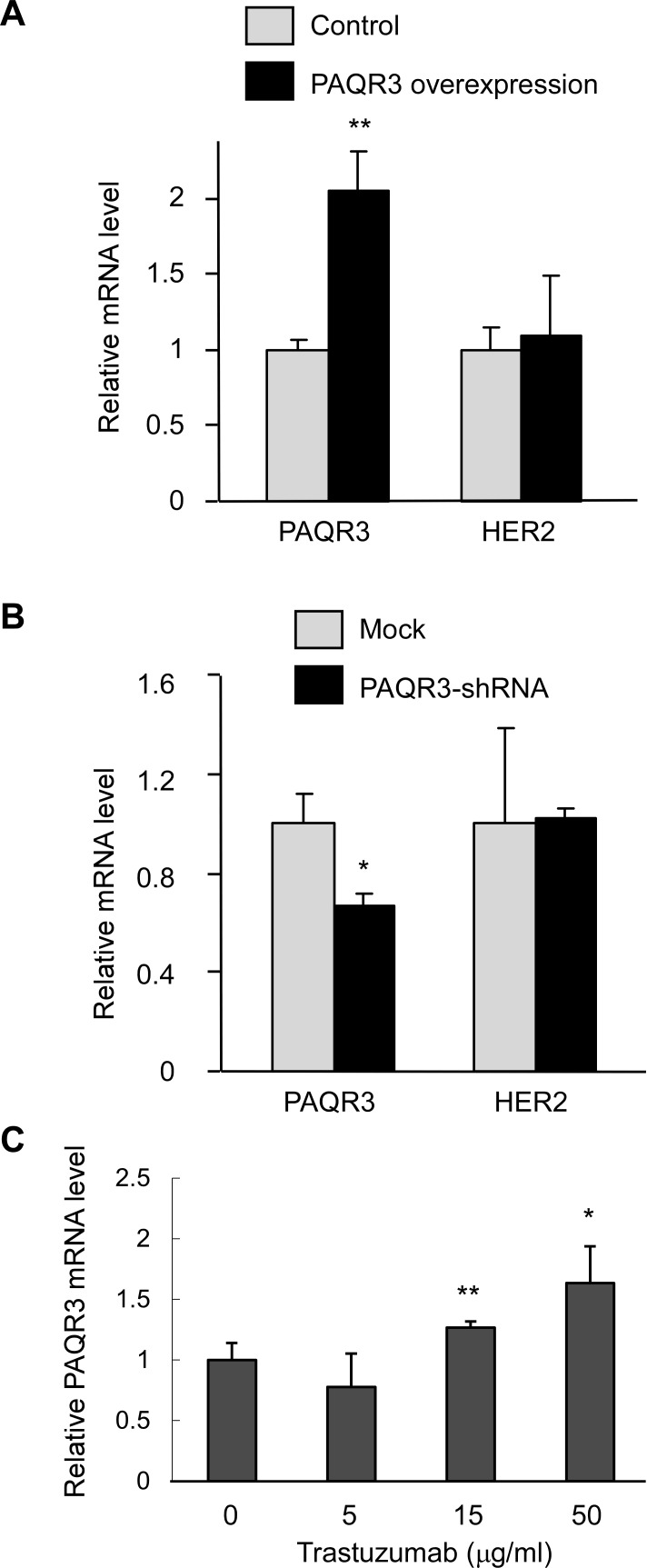
Inhibition of HER2 elevates PAQR3 expression in SKBR3 cells (**A**, **B**) Effect of PAQR3 on HER2 expression. The mRNA levels of PAQR3 and HER2 were measured by quantitative RT-PCR in SKBR3 cells infected with lentivirus that had overexpression of PAQR3 or knockdown of PAQR3 by a PAQR3-specific shRNA. (**C**) Inhibition of HER2 increased PAQR3 expression. SKBR3 cells were treated with various concentrations of trastuzumab for 72 hours before analysis of PAQR3 mRNA level by quantitative RT-PCR. All the data are shown as mean ± SD and * for *P* < 0.05 and ** for *P* < 0.01 as compared to the control group.

Since our clinical data indicate that PAQR3 expression level is inversely correlated with HER2 expression level, we next explored the regulation of HER2 by PAQR3 and *vice versa*. Interestingly, overexpression or knockdown of PAQR3 in HER2-positive SKBR3 cells could not alter the mRNA level of HER2 (Figure 6A and 6B). On the other hand, inhibition of HER2 by trastuzumab could dose-dependently increase PAQR3 expression in these cells (Figure 6C). Collectively, these data suggest that HER2 might regulate PAQR3 expression but PAQR3 has no effect on HER2 expression.

## DISCUSSION

In this study, we have provided evidence that PAQR3 functions as a tumor suppressor in human breast cancers. At the clinical level, PAQR3 expression level was robustly downregulated in human breast cancer samples as compared to the adjacent normal tissues. The expression level of PAQR3 was inversely correlated with HER2 expression level. Furthermore, the expression status of PAQR3 was associated with the survival of the breast cancer patients. At the cellular level, overexpression of PAQR3 negatively regulated cell proliferation, colony formation and migration of human breast cancer cells. Knockdown of PAQR3 in MDA-MB-231 cells upregulated cell growth and migration.

HER2 has served as an important target for the therapy of breast cancers in recent years [3]. Lately, it was found that HER2 and HER3 could cooperatively regulate cancer cell growth and determine sensitivity to TAK-285, an orally bioavailable small molecule inhibitor of HER kinases [24]. Current treatments of breast cancers also include adjuvant or neoadjuvant chemotherapy plus HER2-targeted drugs such as trastuzumab. It was reported that TBK1/IKKepsilon inhibitors might improve the treatment of HER2-postive breast cancers together with anti-HER2 therapy [25]. One the important findings of our study is that the expression level of PAQR3 is closely correlated with HER2 expression. Low expression of PAQR3 is associated with high expression of HER2 (Figure 1C and Table 1). It is currently unknown how PAQR3 expression is associated with HER2 expression at the molecular level. One possibility is that HER2 negatively regulates the expression of PAQR3. Consistently, our preliminary data indicate that modulation of HER2 function could regulate PAQR3 expression in HER2-postive SKBR3 cells (Figure 6). HER2 activation is able to initiate signaling that leads to cell proliferation, survival and migration in cancer cells [5, 6]. PAQR3, on the other hand, can block cell proliferation and survival via negative regulation of Ras/MAPK and PI3K/Akt pathways [15, 19]. Downregulation of PAQR3 by HER2 could result in augmentation of the growth-promoting effect of this growth factor receptor. In other words, the HER2-mediated downregulation of PAQR3 may play a role in a positive feedback to enhance the oncogenic signals of HER2. If this were the case, the expression level of PAQR3 would be crucial for HER2 to execute its oncogenic capacity in cancer cells. It is expected that elevation of the expression or functioning of PAQR3 would attenuate the oncogenic functions of HER2. It is therefore imperative in the future to fully uncover the molecular mechanisms underlying that functional interaction between HER2 and PAQR3 in breast cancer development. Such studies cannot only enhance our understanding about the molecular events occurring during breast cancer formation, but also shed light on future treatment of this deadly disease.

## MATERIALS AND METHODS

### Patients and samples

This study was approved by the Ethics Committee of the Zhejiang Province Cancer Hospital, Hangzhou, Zhejiang, China. All patients provided their full consent to participate in the study. The patients enrolled in this study underwent curative surgery without prior treatments. Tissue specimens were examined separately by two pathologists under double-blinded conditions without prior knowledge of the clinical status of the specimens. The patients' medical records were reviewed to obtain data including age at diagnosis, sex, tumor location, tumor size (diameter), lymph node metastasis, histology, tumor invasion, and TNM stage according to the guidelines of American Joint Committee. Detailed clinical histopathological factors were presented in Table 1. for the measurement of prognosis, we analyzed the clinical data concerning disease-free survival (DFS) and overall survival (OS), defined as the time from surgery to first recurrence or death respectively. All recruited patients had been followed-up periodically until the due date. Clinical follow-up results revealed that the mean follow-up duration was 69 months and the range was 3~109 months.

### Immunohistochemistry analysis

Immunohistochemistry (IHC) was performed using the avidin-biotin-peroxidase complex method with all breast carcinoma samples. All sections were deparaffinised in xylenes and dehydrated through a gradient concentration of alcohol before endogenous peroxidase activity was blocked using 0.5% H_2_O_2_ in methanol for 10 min. After non-specific binding was blocked, the slides were incubated with mouse monoclonal antibodies for ER (1D5) (1:50) and PR (PgR 636) (1:400) in phosphate-buffered saline (PBS) at 4°C overnight in a humidified container respectively. Biotinylated goat anti-rabbit immunoglobulin G (IgG) (1:400; Sigma-Aldrich, St Louis, MO, USA) was incubated with the sections for 1 h at room temperature and detected using a streptavidin-peroxidase complex. The brown color indicative of peroxidase activity was developed by incubation with 0.1% 3,3′- diaminobenzidine (Sigma-Aldrich) in PBS with 0.05% H_2_O_2_ for 5 min at room temperature. Polyclonal HER2 antibody in the Herceptin kit (HercepTest, DAKO, Denmark) was used according to the manufacturer's instructions. Positive controls of known positive breast cancer tissues and negative controls with primary antibody replaced with TBS were run with the patient slides in each run of IHC.

### RNA extraction and real-time RT-PCR analysis of tumor samples

The mRNA expression level of PAQR3 was analyzed by real-time RT-PCR. Total cellular RNAs were extracted using the Trizol (Gibco BRL Life technologies Inc., Rockville, MD, USA) one-step method. A total of 3 μg total RNA was subjected to reverse transcription using M-MLV reverse transcriptase (Promega, San Luis Obispo, CA). The glyceraldehyde phosphate dehydrogenase (GAPDH, TaKaRa Bio, Otsu, Japan) was selected as the internal reference. The primers used in PCR are listed as follows: 5′-TGTCGAAGATGGATGGCATTAGA-3′ and 5′-ACCTGACGCCAGTAGTTATTACA-3′ for PAQR3. 5′-CTGGGCTACACTGAGCACC-3′ and 5′-AAGTGGTCGTTGAGGGCAATG-3′ for GAPDH. The 2^−ΔΔCt^ method was used to calculate relative changes in gene expression. Increased and reduced expressions were defined as the median value of relative gene expression level > 2.0 and < 0.5, respectively.

### Cell culture, transfection and lentiviral transduction

HEK293T and human breast cancer cells MCF7, SKBR3 were cultured in DMEM containing 10% FBS (Invitrogen, Grand Island, NY, USA). Human breast cancer cells MDA-MB-231, MDA-MB-468, MDA-MB-453 were cultured in L15 (Invitrogen) containing 10% FBS. Transient transfection was performed by polyethylenimine (PEI) method. For lentiviral transduction, HEK293T cells (7 × 10^6^) were plated in a 15 cm dish, incubated for 24 h, and then transfected with 15 μg of lentivirus plasmids. After 48 h, the virus-containing medium was filtered through a 0.45 μm filter (Millipore) and collected as first supernatant. Additional medium was added into the plate and the virus-containing medium was filtered and collected as second supernatant after 24h. Both supernatants were centrifuged at 12,000g for 2 h. The supernatant was abandoned and the precipitate was suspended in 100 μl DMEM. For infections, gradient virus-contained DMEM was added into the medium with polybrene (Sigma-Aldrich, 4 μg/ml), and then the culture plates were incubated at 37°C for 6 h and replaced by full medium. After incubation for 36~48 h, the infected cell populations were confirmed by fluorescence microscope for GFP expression to evaluate the virus titer. Target cells were plated in 6-well plates for infections by appropriate virus-contained DMEM.

### RNA isolation and quantitative reverse transcription PCR (RT-PCR) of breast cancer cells

The cells were lysed in Trizol reagent (Invitrogen). Total RNA was purified according to the manufacturer's instructions, then reverse transcribed and synthesized to cDNA using AMV reverse transcriptase (Takara, Kyoto, Japan). The quantification of gene transcripts was determined by real-time qPCR using SYBR Green Realtime PCR Master Mix (Toyobo, Osaka, Japan) and Mx3000P QPCR system (Stratagene, Santa Clara, CA, USA). The PCR primers are 5′-CTCAAGGACAACCCGTACATCAC-3′ and 5′-AAACTTTTGATACACAGCCTGGAC-3′ for PAQR3; 5′-GATCATTGCTCCTCCTGAGC-3′and 5′-ACTCCTGCTTGCTGATCCAC-3′ for β-actin.

### MTT and colony formation assays

For MTT assay, the cells were seeded at a density of 5 × 10^3^ cells/well into a sterile 96-well plate and grown in 5% CO2 at 37°C for 24 h, 48 h and 72 h. Cell viability was measured by 3-(4,5-dimethylthiazol-2-yl)-2,5-diphenyltetrazolium bromide (MTT) assay (from Sigma-Aldrich) as follows: 20 μl of 5 mg/ml MTT was added to each well and incubated with cells for 4 h in an incubator. The formazan was dissolved in 100 μl dimethyl sulfoxide (DMSO) following removal of the medium. Finally the optical density was measured using a spectrophotometer at an absorption wavelength of 570 nm. For colony formation assay, the cells were seeded in to 6-well with 400 cells per well and then cultured for 5 d, followed by crystal violet staining and colony counting.

### Cell migration assays

For transwell assay, the cells were seeded into transwell chambers (Corning, Corning, NY, USA) without FBS and chambers were set into 24-well plates with full medium (with 10% FBS). Cells on lower chambers were fixed in 24 h and stained with crystal violet. Photos were taken under a microscope (Olympus). For wound healing assay, the cells (5 × 10^5^ cells/well) were seeded into a 6-well plate and incubated for 24 h. A wound in each well was then created by scratching the confluent monolayer layer with a yellow tip. After rinsing with PBS three times, cells were incubated with serum-free medium. Photos were taken immediately (t = 0 h) or 24 h later (t = 24 h) under a microscope (Olympus).

### Statistical analysis

SPSS 17.0 statistical software was adopted for analysis of clinical data. Counting data comparisons between groups were subjected to the χ^2^ test and Fisher's exact test. Survival analysis was computed by means of the Kaplan-Meier method and significant levels were assessed by means of the log-rank test. A univariate analysis with the Cox regression model was used to determine prognostic factors, and multivariate analysis with the Cox regression model was used to explore combined effects. Student's *t*-test was used in analysis of cellular data. All results were expressed as the mean ± standard deviation (SD). Values of *P* < 0.05 were considered statistically significant.
